# Optimizing nucleic acid delivery using PF14 peptide and lipid nanoparticle systems

**DOI:** 10.1016/j.ijpx.2026.100646

**Published:** 2026-08-21

**Authors:** Delia Daniele, Zaw Myo Hein, Osama Saher, Ibrahim El-Serafi

**Affiliations:** a Department of Laboratory Medicine, Karolinska Institute, Stockholm, Sweden; b Department of Cellular Therapy and Allogeneic Stem Cell Transplantation (CAST), Karolinska University Hospital Huddinge and Karolinska Comprehensive Cancer Center, Stockholm, Sweden; c Karolinska ATMP Center, Karolinska Institutet, ANA Futura, Stockholm, Sweden; d Basic Medical Sciences Department, College of Medicine, Ajman University, Ajman, United Arab Emirates; e Department of Pharmaceutics and Industrial Pharmacy, Faculty of Pharmacy, Cairo University, Cairo, Egypt

**Keywords:** Nucleic acid delivery, Cell-penetrating peptides, PF14, Lipid nanoparticles, Gene therapy, RNA therapeutics, Drug delivery systems

## Abstract

Nucleic acid–based therapeutics are a rapidly expanding class of precision medicines capable of directly modulating gene expression. However, their clinical application is limited by challenges in cellular delivery, including large molecular size, hydrophilicity, and susceptibility to enzymatic degradation. To address these barriers, advanced delivery platforms such as cell-penetrating peptides and lipid nanoparticles (LNPs) have been developed.

This study evaluates two delivery systems: the cell-penetrating peptide PepFect-14 (PF14) and LNP formulations, for enhancing nucleic acid delivery and cellular uptake. PF14 facilitates intracellular transport through peptide–nucleic acid complex formation, while LNPs protect cargo from degradation and can promote endosomal escape. Their performance was assessed under different conditions using luciferase-based reporter systems in HeLa cells.

PF14-mediated delivery of the splice-switching oligonucleotide ON-705 in HeLa 705 cells showed efficient splice correction, with activity increasing in a dose- and molar ratio- dependent manner, reaching a plateau at a 1:10 ratio. Delivery efficiency was significantly influenced by formulation conditions, with Opti-MEM outperforming standard media and sugar-based buffers. Polymer excipients also affected activity as PVA18, PVA40, and PVP40 enhanced performance, while low molecular weight PVP reduced efficacy. In parallel, LNP-mediated delivery of luciferase mRNA in wild-type HeLa cells demonstrated robust, dose-dependent protein expression. Both systems maintained high cell viability (80–100%).

Overall, these findings highlight the importance of formulation optimization in improving nucleic acid delivery and provide practical insights for enhancing peptide- and lipid-based therapeutic platforms.

## Highlights

• Formulation parameters strongly influenced PF14-mediated oligonucleotide delivery.
• PF14 enhanced splice-switching activity in a dose- and ratio-dependent manner.
• Buffer and polymer selection critically affected nanoparticle performance.
• LNPs achieved robust dose-dependent mRNA expression in HeLa cells.
• PF14 and LNP systems maintained high cell viability across tested conditions.

## Graphical abstract

**Figure:**
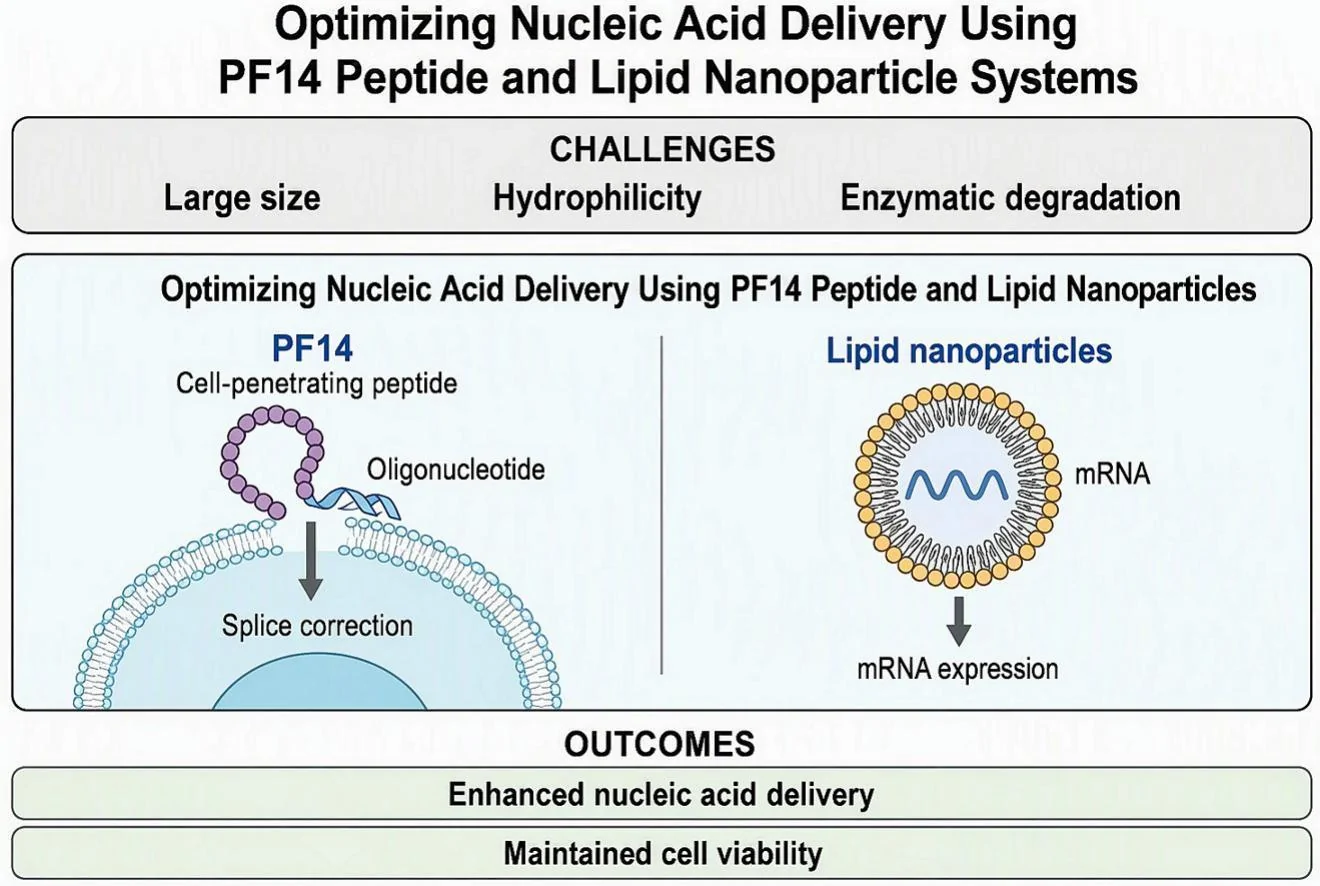

## 1 Introduction

Nucleic acid-based therapeutics hold significant promise for the treatment and management of a diverse array of diseases. This class of drugs encompasses various modalities, including small interfering RNAs (siRNAs), microRNAs (miRNAs), antisense oligonucleotides (ASOs), splice-switching oligonucleotides (SSOs), and messenger RNAs (mRNAs). While mRNA therapeutics primarily aim to induce or restore protein expression, oligonucleotide-based approaches facilitate gene silencing, splice modulation, or gene activation. These advanced strategies enable the targeting of a broader spectrum of biological pathways and molecular mechanisms, extending beyond the reach of conventional pharmaceutical modalities (Roberts et al., 2020).

Conventional small-molecule drugs achieve their therapeutic effects primarily by binding to specific protein pockets. However, many potential targets lack such well-defined binding surfaces, limiting the scope of these compounds. In contrast, nucleic acid-based therapeutics overcome this limitation by targeting cellular DNA or RNA through sequence-specific recognition and hybridization, enabling the precise modulation of virtually any genetic sequence. The primary challenge associated with nucleic acid therapeutics lies in their pharmacokinetics. Unlike small lipophilic molecules, which readily penetrate cells, nucleic acids are typically larger and more hydrophilic, presenting significant barriers to cellular uptake (Smith and Zain, 2019). Moreover, RNAs are highly susceptible to destruction by nucleases or hydrolases in blood or body fluids (Soutschek et al., 2004). In some cell types, naked mRNA can be spontaneously internalized by scavenger-receptor-mediated endocytosis; however, it will later accumulate in the lysosome, from which only small amounts can leak into the cytoplasm (Sahin et al., 2014).

The delivery challenge is particularly pronounced for small oligonucleotides such as ASOs: on average < 1% of ASOs reach the correct cellular compartment. Therefore, significantly enhancing cell-specific delivery of ASOs is extremely important for therapeutic efficacy (Godfrey et al., 2017).

An ideal RNA delivery system should effectively encapsulate and protect RNAs from degradation by nucleases during systemic delivery, ensuring prolonged circulation in the bloodstream while avoiding rapid clearance by the kidney and phagocytosis by the liver or spleen. It should also enhance targeted accumulation within specific tissues or organs, promote efficient cellular uptake, and bypass lysosomal degradation during intracellular trafficking to ensure the release of RNAs into the cytoplasm (Yan et al., 2022).

Multiple strategies have been developed to deliver various nucleic acid drugs to target cells. In particular, mRNA delivery has evolved through different delivery vectors over the years. First-generation mRNA delivery systems used complexation with protamine, polyethyleneimine (PEI), and cationic liposomes (Schafer et al., 2010). Second-generation delivery systems were based mainly on biodegradable ionizable polymers used as scaffolds (Chen et al., 2018). However, many of these materials failed in clinical research due to high toxicity, complex structures, and uncontrollable polymerization (Bitounis et al., 2024; Ismail and Chou, 2025; Yang et al., 2023).

Third-generation mRNA delivery systems, based on lipid nanoparticles (LNPs), have addressed these challenges with features such as excellent biocompatibility, high nucleic acid encapsulation efficiency, effective transfection, deep tissue penetration, intelligent drug release, low off-target effects, and minimal cytotoxicity and immunogenicity. LNPs are spherical vesicles composed of ionizable lipids, which are less toxic than permanently positively charged lipids like liposomes. They remain neutral at physiological pH, reducing non-specific interactions with serum proteins, but become positively charged at low pH, enhancing their binding to negatively charged mRNA. This property improves mRNA stability, promotes endosomal escape, and facilitates efficient transfection in vivo (Yang et al., 2022).

Other components of these LNPs are neutral phospholipids that facilitate cell binding and destabilize endosomes, cholesterol which stabilizes LNP structure, and the polyethylene glycol (PEG) chains attached to lipid moieties, PEGylated lipids, which reduce particle aggregation (Yang et al., 2020). LNP delivery of mRNA involves the apolipoprotein E (ApoE)-dependent pathway. When the LNP:mRNA complex reaches the cell membrane, ionizable lipids in the LNPs fuse with the negatively charged cell membrane, allowing uptake through endocytosis. Inside the lysosome, the acidic environment causes ionizable lipids to protonate, creating hexagonal structures that break apart the LNP bilayer, releasing mRNA into the cell (Yang et al., 2022).

Concerning delivery of smaller oligonucleotides, various strategies have been developed including bioconjugation, lipid and carbohydrate conjugates, antibody and aptamer conjugates, peptide conjugates, nanocarriers, lipoplexes, lipid-based systems, and exosome-based therapeutics (Juliano, 2016). While liposomes protect ASOs, they are destabilized by serum proteins: opsonins tag them for phagocytosis (Bozzuto and Molinari, 2015), while lipoproteins strip lipids, releasing their cargo (Zelphati et al., 1998). PEGylation reduces protein interactions and immunogenicity by creating a stealth effect (Nag and Awasthi, 2013), although limitations exist due to PEG non-biodegradability and antibody formation (Abu Lila et al., 2013; Hoang Thi et al., 2020; Schottler et al., 2016). Alternatives such as poly(glycerols), poly(oxazolines), poly(zwitterions), and stimuli-sensitive liposomes are under exploration (Estephan et al., 2011; Lee and Thompson, 2017; Paliwal et al., 2015).

A promising strategy for small oligonucleotide (ON) delivery uses cell-penetrating peptides (CPPs), which are cationic or amphipathic peptides generally up to 30 amino acids capable of delivering diverse nucleic acid cargoes into cells (Boisguerin et al., 2015; Pooga and Langel, 2015). The initial CPPs were derived from protein sequences known for their translocation properties. Over time, these peptides were engineered and combined with domains from diverse naturally occurring proteins to enhance or modify their functionality. Advances in the understanding of CPP characteristics have facilitated the design of peptides with entirely synthetic sequences, utilizing computational algorithms to predict amino acid arrangements with translocating capabilities (Hansen et al., 2008). CPPs carrying cargo molecules are primarily internalized into cells via energy-dependent mechanisms, including various types of endocytosis such as macropinocytosis, clathrin-mediated, and caveolae-mediated pathways. The specific endocytic pathway utilized depends on factors such as the properties and concentration of the cargo molecules, as well as the cell type or tissue being studied. These pathways may function simultaneously or alternatively, with one compensating for the inhibition of another (Margus et al., 2013). Following endocytosis-mediated internalization, CPPs are typically trapped within *endo*-lysosomal compartments, a process known as endosomal entrapment. This step is a major barrier to the bioavailability of CPPs (El-Sayed et al., 2009). Effective endosomal escape is therefore critical for CPPs to achieve their intended activity. To address this challenge, research has focused on strategies to enhance endosomal escape. These strategies include modifying CPPs with hydrophobic compounds (e.g., fatty acids or cholesterol) to improve membrane interactions, incorporating fusogenic peptide sequences derived from viral endosomolytic domains (e.g., the HA2 domain of influenza virus A), and adding protonatable domains (e.g., chloroquine analogs or histidines) to buffer the endo-lysosomal environment. However, achieving a balance between efficient endosomal escape and minimizing cellular toxicity remains a key challenge (Lehto et al., 2016). CPPs can deliver ONs through two main methods: covalent conjugation or nanoparticle formation. In the covalent conjugation approach, CPPs and ONs are chemically linked, resulting in a molecule with a well-defined structure and stoichiometry (Jarver et al., 2012). However, this method is generally limited to charge-neutral ONs, as charged ONs are difficult to purify and tend to form complexes due to electrostatic interactions between the anionic ONs and cationic CPPs (Lehto et al., 2016). Moreover, this strategy may alter the biological activity of conjugates (Guidotti et al., 2017). In contrast, the nanoparticle-based approach is more versatile and can accommodate larger nucleic acid molecules, such as plasmids (Deshayes et al., 2008). Here, nanoparticles form through electrostatic and hydrophobic interactions between the anionic nucleic acids and cationic CPPs. However, these nanoparticles are less defined and often unstable, prone to dissociation in biological fluids, and have limited endosomal escape efficiency. To address these issues, additional modifications are often necessary to stabilize the nanoparticles and improve their functionality (Lehto et al., 2016). An example of a modified CPP is PepFect-14 (PF14), a derivative of the stearyl-transportan10 (stearyl-TP10) peptide (Mae et al., 2009). PepFect peptides are an intriguing group of amphipathic CPPs that have shown delivery potential for a wide variety of nucleic acid-based cargoes, including SSOs, siRNAs, and plasmids (Lehto et al., 2016). In PF14, lysines and isoleucines are replaced with ornithines and leucines, a modification designed to enhance transfection efficiency compared to poly-l-lysine-based systems. This improvement is attributed to the higher DNA-binding affinity of poly-l-ornithines and their ability to form more stable complexes at lower charge ratios (Ramsay and Gumbleton, 2002).

In this study, we investigated strategies to improve the therapeutic efficiency of two delivery systems: PF14 complexes with SSOs and LNPs for mRNA delivery. We aimed to identify conditions that maximize the therapeutic potential of both PF14-SSO complexes and LNP-based mRNA delivery systems.

## 2 Materials and methods

### 2.1 Cell Lines and culture

The HeLa pLuc 705 stable cell line (kindly gifted by Professor Ryszard Kole, The University of North Carolina, USA), herein referred to as HeLa 705, was used to evaluate SSO delivery efficacy. HeLa 705 cells contain a luciferase gene interrupted by a mutated β-globin intron 2, causing aberrant splicing and aberrant luciferase expression. Wild-type HeLa cells (purchased from ATCC, Manassas, VA, USA) were used for mRNA delivery studies with LNPs. All cultures were maintained in DMEM with 10% FBS and Antibiotic-Antimycotic mix under standard conditions (37 °C, 5% CO_2_).

### 2.2 Preparation of PepFect-14-SSO complexes and LNPs

PF14, a stearylated cell-penetrating peptide (Pepscan, The Netherlands), was complexed at various molar ratios (MR) with the splice-switching oligonucleotide ON-705. The 18-mer splice-switching ON with the sequence (5’-CCUCUUACCUCAGUUACA) was synthesized at GE Healthcare and had a fully modified phosphorothioate backbone and 2’-*O*-methyl modified ribose sugar units. Complexes were prepared in different buffers including Opti-MEM, HBG (20 mM HEPES +5% glucose), SH (20 mM HEPES +10% sucrose), DMEM, and RPMI (Invitrogen, Sweden). Complexation typically involved incubation at room temperature for 20 min to allow nanoparticle formation.

LNPs containing luciferase mRNA were prepared as described previously (Ueda et al., 2024). Briefly, the lipid phase was prepared by dissolving MC3, DSPC, cholesterol, and DMG-PEG in ethanol at a molar ratio of 50:10:38.5:1.5, corresponding to the composition of clinically approved LNP formulations. The total lipid concentration was maintained at 4 mM and LNPs were stored at −80 °C until use (Ueda et al., 2024). LNP stocks were thawed on ice and diluted in Opti-MEM immediately before adding cells, to the desired final mRNA concentration.

### 2.3 Evaluation of added polymer effects

Polymeric excipients were evaluated for both delivery systems. Polyvinyl alcohol (PVA; 18 and 40 kDa) and polyvinylpyrrolidone (PVP; 10 and 40 kDa) (Sigma–Aldrich, Stockholm, Sweden) were each prepared as 10% (*w*/*v*) stocks in 12.6% sucrose. For PF14 complexes, polymer stocks were added after the initial 20 min complexation step and incubated for an additional 20 min at room temperature (Daralnakhla et al., 2021; Saher et al., 2019; Saher et al., 2018). For LNPs, polymer stocks were added to thawed, Opti-MEM -diluted LNPs and incubated for 20 min at room temperature. In both systems, polymers were added post-formulation and therefore did not affect mRNA or oligonucleotide encapsulation (Ma et al., 2025; Periyasamy et al., 2023).

### 2.4 Cell treatment protocol

HeLa 705 cells were treated with PF14-SSO complexes and wild-type HeLa cells with LNP formulations under serum-free conditions, predominantly in Opti-MEM or specified buffers. In all experiments, 10 μL of PF14-complex or LNP suspension was added to cells growing in 100 μL of DMEM culture medium. This minimal medium perturbation ensured that differences in transfection efficiency reflected complex performance rather than buffer-induced effects on cell viability. Treatments were applied for 24 h before analysis (Bazaz et al., 2021; Daralnakhla et al., 2021; Periyasamy et al., 2023; Saher et al., 2019; Saher et al., 2018).

### 2.5 Luciferase reporter assay

Post-treatment, cells were lysed, and luciferase activity was measured using standard luminometric assays. Activity was normalized to untreated control samples and reported as fold change (Daralnakhla et al., 2021; Hande et al., 2019; Periyasamy et al., 2023).

### 2.6 Cellular uptake and fluorescence microscopy

Alexa Fluor 568-labelled ON705 was complexed with PF14 at peptide-to-oligonucleotide molar ratios ranging from 1:1 to 1:20, with or without poly(vinyl alcohol) (PVA) or poly(vinylpyrrolidone) (PVP). Complexes were applied to wild-type HeLa cells at a final ON705 concentration of 100 nM. Live-cell fluorescence images were acquired after 4 and 24 h using a ZOE™ Fluorescent Cell Imager (Bio-Rad) to qualitatively assess cellular uptake (Daralnakhla et al., 2021; Ezzat et al., 2011; Saher et al., 2019).

### 2.7 Characterization of nanoparticles

Freshly prepared complexes were subjected to particle size measurement using a ZetaView® PMX system (Particle Metrix, Meerbusch, Germany). Complexes, with or without excipients, were prepared in specified buffer and diluted prior to measurement in 0.1× PBS (Invitrogen, Stockholm, Sweden). Particle size and zeta potential were determined for each sample. Measurements were performed at least three times per sample (Bazaz et al., 2021; Ma et al., 2025)**.**

### 2.8 Gel retardation assay

The efficiency of ON-705/PF14 complex formation was assessed by gel retardation. Complexes were prepared as described in Section 2.1, at a final ON-705 concentration of 3 μM in a 10 μL reaction volume. For molar ratio screening, complexes were prepared at MRs of 1:1, 1:2, 1:3, 1:4, 1:10, and 1:20 in Opti-MEM and incubated for 20 min at room temperature. For polymer screening, ON-705/PF14 complexes were prepared at MR 1:10, then PVA18, PVA40, PVP10, or PVP40 (10% *w*/*v* in 12.6% sucrose) was added and incubated for a further 20 min at room temperature. Naked ON-705 was included as reference. Samples were loaded onto 2% agarose gels in 1× Tris-acetate-EDTA (TAE) buffer, stained with SYBR Green II (Invitrogen, Molecular Probes) at 1:10,000 dilutions. Gels were imaged using a ChemiDoc MP Imaging System (Bio-Rad) (Ezzat et al., 2011; Saher et al., 2018).

### 2.9 Cell viability

The WST-1 assay was performed according to the manufacturer's recommendation. Cells were seeded and transfected in 96-well plates. One day after treatment, the medium was replaced with fresh medium supplemented with WST-1 reagent (Sigma–Aldrich, Stockholm, Sweden) (1:10 dilution). The cells were then incubated for 2 h at 37 °C in a humidified incubator with 5% CO_₂_. Absorbance was measured at 450 nm with a reference wavelength of 650 nm using a SpectraMAX i3x plate reader (Molecular Devices, San Jose, CA, USA). Results were expressed as the percentage ratio of the absorbance at 450 nm of treated cells relative to untreated cells (Saher et al., 2018).

### 2.10 Data analysis

All assays were performed in triplicate, with mean ± SEM calculated. Statistical analyses (One-way ANOVA) were conducted with GraphPad Prism software, and using Bonferroni as a post-hoc test. Significance thresholds set at *p* < 0.05.

## 3 Results

### 3.1 Increasing molar ratios of ON-705/PepFect-14 enhances efficacy

To evaluate the efficiency of the CPP PF14 in delivering SSOs we chose a system of HeLa pLuc 705, herein called HeLa 705. HeLa 705 is a cell line stably transfected with a luciferase-encoding gene interrupted by a mutated β-globin intron.

**Fig. 1:**
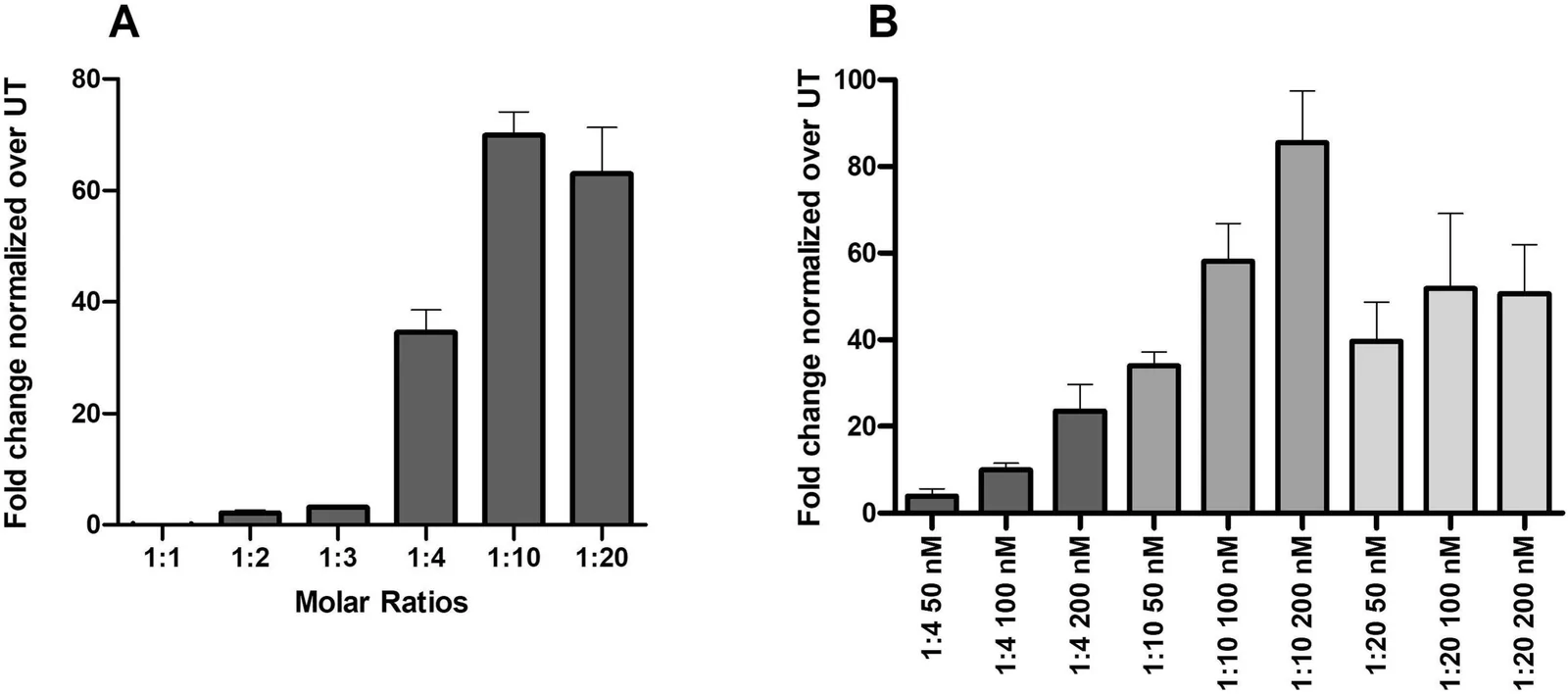
Luciferase Assay values after treatment with ON-705/PF14 in Opti-MEM. Cells were treated with ON-705/PF14 nanoparticles in serum-free Opti-MEM media for 24 h. Thereafter, luciferase expression was measured by luminometric analysis. Splice-switching activity is presented as a fold increase in luciferase activity over untreated cells. Values indicate the mean of at least three independent experiments measured in triplicates (mean ± SEM, *N* ≥ 3). (A) The nanoparticles were formulated at six different MRs using 100 nM of ON-705. (B) The nanoparticles were formulated at three MRs using 50 nM, 100 nM, and 200 nM of ON-705. This elicited a dose-dependent response. Statistical analyses (One-way ANOVA) were conducted with GraphPad Prism software, and using Bonferroni as a post-hoc test.

2. This mutation results in an aberrant splicing of luciferase pre-mRNA, causing the synthesis of non-functional luciferase. By masking the aberrant splice site with a splice-switching ON it is possible to restore the correct mRNA splicing and rescue the luciferase activity (Mae et al., 2009). PF14 was thus used to deliver an SSO, herein named ON-705, capable of restoring the original splicing pattern of the gene and rescuing the expression of the protein. Based on the literature review, the dosage selected for this preliminary assay was 100 nM. Following the complexation of the ON-705 with PF14, the treatment was administered to cells and incubated for 24 h. Subsequent luciferase assays demonstrated the restoration of the luciferase signal, indicative of successful splicing correction, across all treated cell samples. A range of MR between SSOs and PF14 was evaluated (Fig. 1A). The data revealed that lower MRs in serum-free Opti-MEM media produced minimal effects, whereas increasing the peptide-to-oligonucleotide ratio significantly enhanced treatment efficacy, as reflected in the luciferase signal. These findings highlight the critical role of PF14 in facilitating efficient cellular delivery of SSOs.

Notably, a plateau effect was observed at an MR of 1:10, beyond which further increases in PF14 did not improve the restoration of the luciferase signal. This suggests that the delivery system has an upper limit in its capacity to transport SSOs into cells, corresponding to a maximum achievable level of luciferase signal restoration. These results underscore the importance of optimizing the peptide-to-oligonucleotide ratio to achieve maximum therapeutic efficiency.

### 3.2 The effects of ON-705 are dose-dependent

To further understand the relationship between the therapeutic effect elicited by the splice-switching oligonucleotide and the dose administered to the cells, we conducted a dose-response study. Specifically, increasing concentrations of ON-705 were tested, with final concentrations of 50 nM, 100 nM, and 200 nM in the cell culture wells. Each dose was complexed with PF14 Opti-MEM at three distinct MRs: 1:4, 1:10, and 1:20. This approach allowed us to assess the combined impact of SSO dose and peptide-to-oligonucleotide ratio on treatment efficacy. The results demonstrated a clear dose-dependent increase in the luciferase signal, indicative of enhanced splicing correction with higher ON-705 concentrations (Fig. 1B). Within each molar ratio tested, the signal exhibited a consistent upward trend as the SSO dose increased. However, at the highest dose tested (200 nM), the increasing trend between different MRs was not maintained. Specifically, no additional enhancement was observed beyond the MR of 1:10, providing further evidence of a plateau effect.

### 3.3 Physicochemical characterization of the ON-705/PF14 complexes

**Fig. 2:**
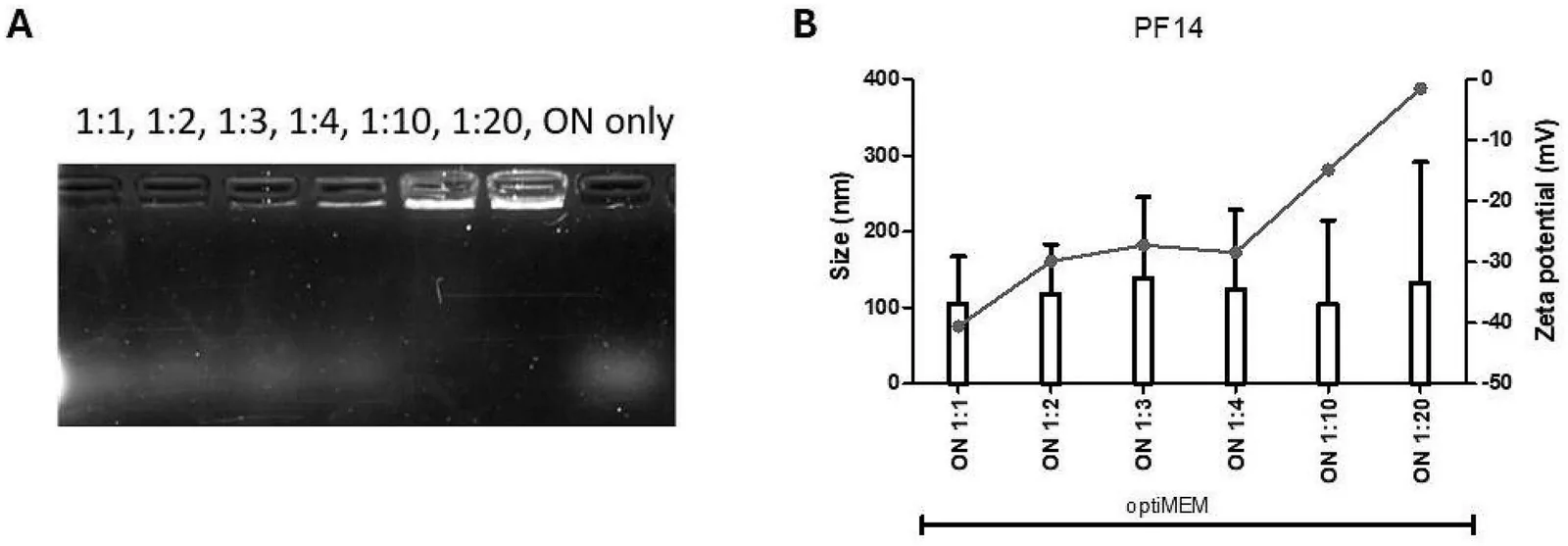
Characterization of the complexation efficiency and the physicochemical properties of the ON-705/PF14 formulations. (A) ON-705/PF14 nanoparticles were formulated at six MRs in serum-free Opti-MEM media with a final ON-705 concentration of 3 μM in a 10 μL reaction volume; to assess the complexation efficiency, these formulations were then loaded on a 2% agarose gel in 1× Tris-acetate-EDTA (TAE) buffer, and then stained with SYBR Green II. Naked ON-705 was included as reference. (B) ON-705/PF14 nanoparticles were formulated at six MRs in serum-free Opti-MEM media, and particle size and zeta potential were measured using a ZetaView PMX system. (For interpretation of the references to colour in this figure legend, the reader is referred to the web version of this article.)

To verify the complexation efficiency of ON-705 by PF14 at different MRs, a gel retardation assay was performed (Fig. 2A). At low MRs (1:1 to 1:4), the majority of ON-705 band was visible at the migration front indicating partial complexation. With increasing MR, the free ON band progressively faded and was no longer detectable at MR 1:10 and 1:20, and strong signals were observed in the wells suggesting a near-complete complexation at the optimal MR identified in the biological assays, consistent with the plateau effect observed.

Additionally, the different MRs were analyzed for size and charge, using Zetaview, to further understand the properties of different formulations (Fig. 2B). Because NTA tracks and sizes individual particles, the reported standard deviation reflects the actual breadth of the particle size distribution and is systematically larger than the intensity-weighted dispersity obtained by DLS. The peptide/ON complexes formed nanoparticles with sizes generally around 100–200 nm, which was fairly consistent across all MRs, while the electrical charge showed progressive increase, the more PF14 was used in the complexes. The MR of ON 1:10 PF14 formulation showed a mean particle size of 103.8 ± 110.7 nm with a zeta potential of −14.92 ± 0.71 mV.

These findings suggest that the observed plateau after the MR 1:10 may reflect the saturation of ON complexation by PF14, to effectively transport SSOs into cells or a maximal level of splicing correction achievable under these conditions. This highlights the importance of optimizing both the SSO dose and the peptide-to-oligonucleotide ratio to balance therapeutic efficacy and system limitations.

### 3.4 Fluorescence microscopy confirms PF14-mediated oligonucleotide uptake

Representative fluorescence microscopy images confirmed intracellular uptake of Alexa Fluor 568-labelled ON705 delivered by PF14 complexes (Supplementary Fig. S1). Cellular fluorescence increased with increasing PF14:ON705 ratio and reached a plateau at a ratio of 1:10, while no further increase was observed at 1:20, consistent with the luciferase and nanoparticle characterization data.

The effect of polymeric excipients was evaluated at the optimal 1:10 ratio. As shown in Supplementary Fig. S2, formulations containing PVA or PVP exhibited fluorescence patterns comparable to those of excipient-free complexes, indicating no apparent effect on cellular uptake under the tested conditions.

### 3.5 The formulation buffer for ON-705/PepFect-14 influences the efficacy of the treatment

**Fig. 3:**
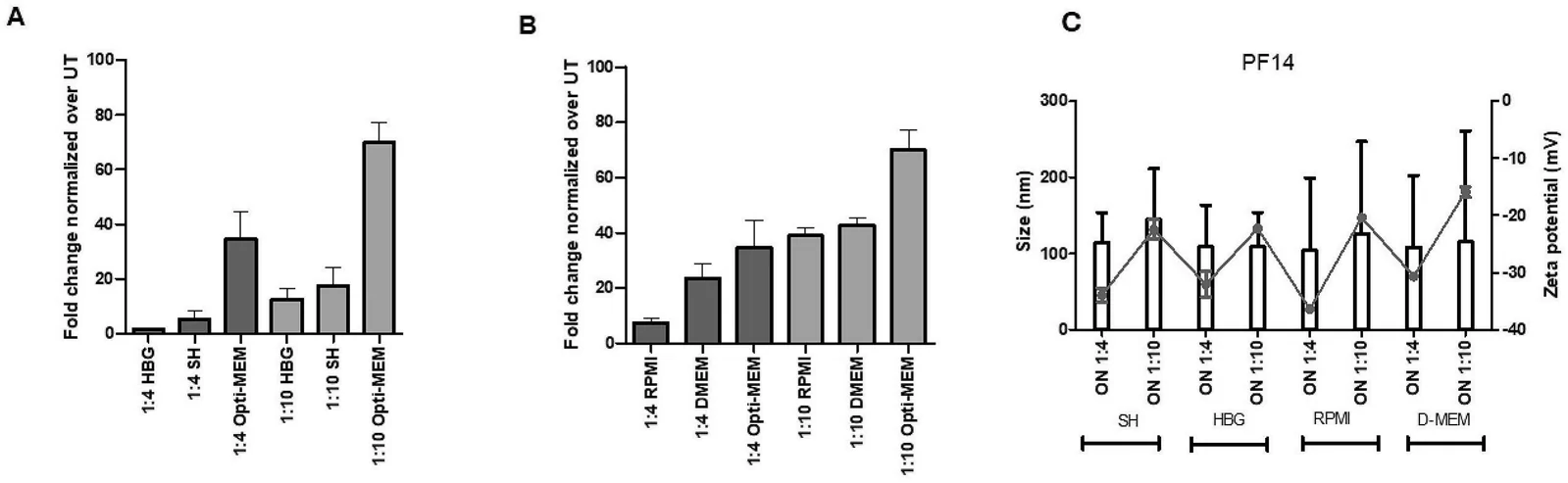
Luciferase Assay values after treatment with ON-705/PF14 in different buffers and culturing media. Cells were treated with ON-705/PF14 nanoparticles formulated at two MRs using 100 nM of ON-705 in different buffers for 24 h. Thereafter, luciferase expression was measured by luminometric analysis. Splice-switching activity is presented as a fold increase in luciferase activity over untreated cells. (A) The efficiency of Opti-MEM formulation was compared with two sugar-based buffers: HBG (20 mM HEPES +5% glucose) and SH (20 mM HEPES +10% sucrose). (B) The efficiency of the Opti-MEM formulation was compared with two culturing media, DMEM and RPMI. (C) ON-705/PF14 nanoparticles were formulated at two different MRs in serum-free Opti-MEM or other indicated buffer or culturing media, and size and charge were measured using a ZetaView PMX system. Values indicate the mean of at least three independent experiments measured in triplicates (mean ± SEM, *N* ≥ 3). Statistical analyses (One-way ANOVA) were conducted with GraphPad Prism software, and using Bonferroni as a post-hoc test.

To further optimize the delivery and efficacy of the SSO-PF14 complexes, we investigated the influence of different formulation buffers on the restoration of the luciferase signal. The choice of buffers is a critical factor in ensuring the stability and bioavailability of nucleic acid formulations, as it can impact complex formation, cellular uptake, and overall bioactivity. Initially, we compared the effects of two sugar-based buffers: HBG buffer (20 mM HEPES with 5% d-glucose) and SH buffer (20 mM HEPES with 10% sucrose). However, formulations prepared using these buffers demonstrated reduced efficacy compared to the standard Opti-MEM medium, with diminished restoration of the luciferase signal observed in treated cells (Fig. 3A). This suggested that neither HBG nor SH buffer could improve the performance of the SSO-PF14 complexes under the tested conditions.

Given these findings, we shifted our focus to testing cell culture media as alternative formulation buffers. Two widely used media, DMEM and RPMI, were evaluated for their potential to enhance treatment efficacy. Formulations prepared in these media showed improved effects compared to the sugar-based buffers, as indicated by higher luciferase signal restoration. However, neither DMEM nor RPMI outperformed the original Opti-MEM formulation (Fig. 3B).

Furthermore, physicochemical characterization of complexes prepared in the different buffers revealed comparable particle sizes (∼100–145 nm) across all conditions but pronounced differences in surface charge (Fig. 2B; Fig. 3C). At MR 1:20, complexes formed in Opti-MEM (Fig. 2B) displayed the least negative zeta potential (−1.58 mV), whereas those prepared in HBG, SH, RPMI, and DMEM retained more negative surface charges (−15.9 to −22.5 mV). A similar pattern was observed between Opti-MEM and yesother media at MR 1:4 (Fig. 2B; Fig. 3C). This suggests that Opti-MEM supports more efficient peptide–oligonucleotide charge neutralization, which likely facilitates cellular uptake and explains the higher transfection efficacy observed in this medium.

These results suggest that Opti-MEM provides a more suitable environment for ON-705/PF14 complex formation and delivery compared to the other tested buffers. This underscores the importance of selecting an appropriate formulation buffer to maximize the therapeutic efficacy of nucleic acid-based treatments. Future studies may explore additional buffer compositions or buffer modifications to further optimize delivery systems.

### 3.6 PVA and PVP polymers influence the efficacy of the treatment

In the next stage of our investigation, we evaluated the impact of incorporating polymers into the ON-705/PF14 delivery system to enhance its effectiveness. PVA and PVP were selected because of their established use as pharmaceutical excipients with favorable biocompatibility, their ability to stabilize nanoparticle formulations against aggregation, and prior reports of their beneficial effects on nucleic acid delivery systems (Daralnakhla et al., 2021; Saher et al., 2019; Saher et al., 2018) The polymers selected for this study were PVA and PVP, which were prepared at a concentration of 10% in a 12.6% sucrose solution dissolved in distilled water. To assess the potential influence of molecular weight (MW) on the delivery system's performance, we tested both low and high MW variants of these polymers.

**Fig. 4:**
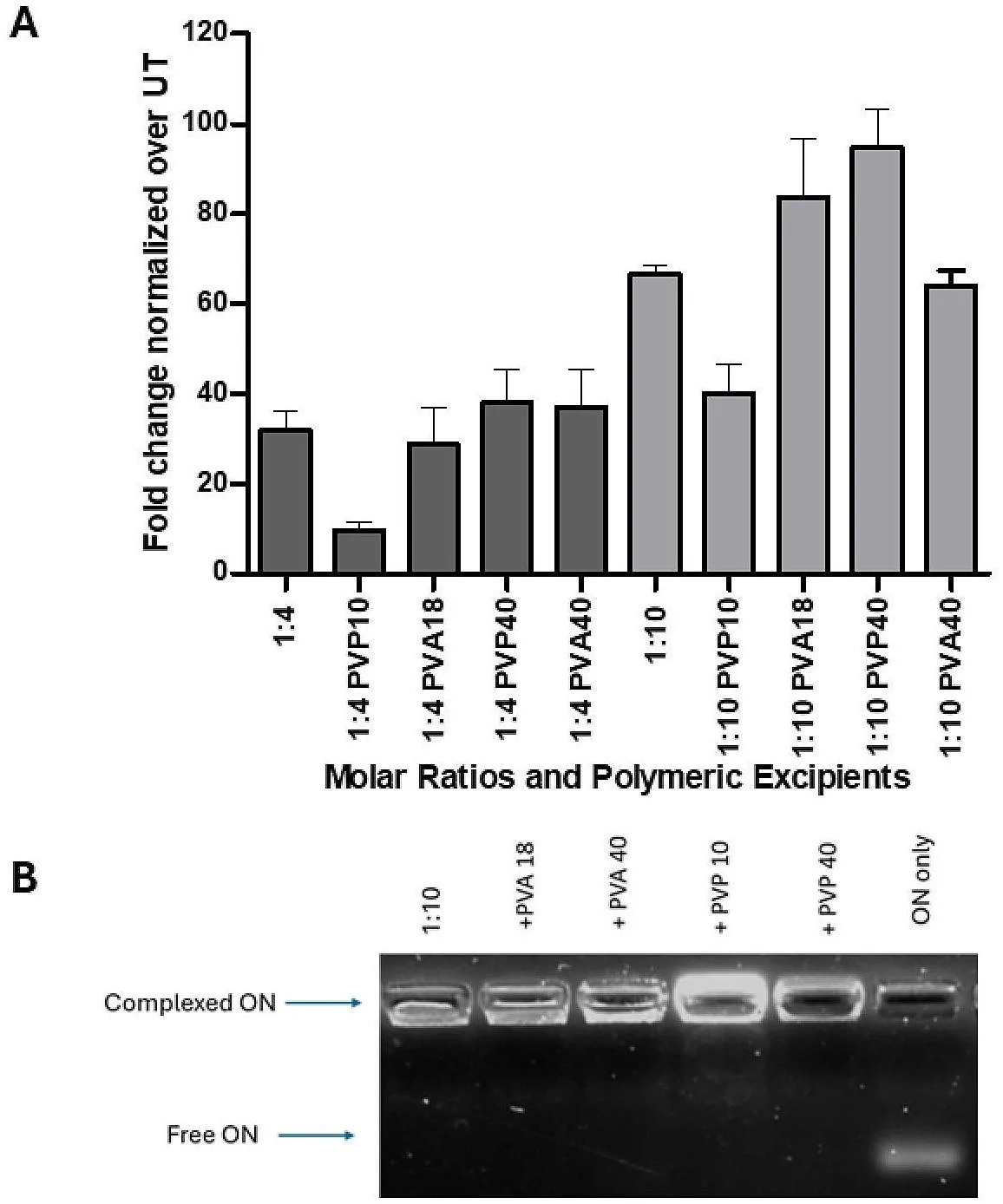
Addition of different polymers to the ON-705/PF14 formulations. (A) ON-705/PF14 nanoparticles were formulated at two MRs in serum-free Opti-MEM media and then supplemented with different polymers; these formulations were then used to treat cells using 100 nM of ON-705 for 24 h. Thereafter, luciferase expression was measured by luminometric analysis. Splice-switching activity is presented as a fold increase in luciferase activity over untreated cells. Values indicate the mean of at least three independent experiments measured in triplicates (mean ± SEM, *N* ≥ 3). Statistical analyses (One-way ANOVA) were conducted with GraphPad Prism software, and using Bonferroni as a post-hoc test. (B) ON-705/PF14 nanoparticles were formulated at 1:10 MR in serum-free Opti-MEM media with a final ON-705 concentration of 3 μM in a 10 μL reaction volume, and then supplemented with different polymers; to assess the complexation efficiency, these formulations were loaded on a 2% agarose gel in 1× Tris-acetate-EDTA (TAE) buffer, and then stained with SYBR Green II. (For interpretation of the references to colour in this figure legend, the reader is referred to the web version of this article.)

Specifically, the low MW variants included PVP (10 kDa) and PVA (18 kDa), while the high MW variants were PVP and PVA (40 kDa). The formulations were evaluated for their ability to enhance the restoration of the luciferase signal in treated cells. Interestingly, the inclusion of low MW PVP (10 kDa) in the formulation was associated with a reduction in the luciferase signal at both MR 1:4 and 1:10, suggesting a detrimental effect on treatment efficacy. In contrast, the beneficial effect of polymer addition depended on both polymer identity and molar ratio: at MR 1:4, PVA40 (high MW) enhanced signal restoration, whereas at MR 1:10, PVA18 (low MW) and PVP40 (high MW) increased signal restoration (Fig. 4A).

In order to assess if the polymers were affecting the complexation of the nanoparticles, we performed a gel retardation assay. We chose MR 1:10 which was formulated and then mixed with the different polymers, before loading on a 2% gel (Fig. 4.B). In all the tested 1:10 complexes, the addition of the polymers (PVA18, PVA40, PVP10, or PVP40) did not release ON from the wells, which was confirmed by the absence of any free ON bands, thus confirming that polymer incorporation does not displace bound oligonucleotide or destabilize the complexes.

These findings highlight that polymer type, molecular weight, and the peptide-to-oligonucleotide ratio together determine the performance of the ON-705/PF14 delivery system, underscoring the need for careful selection of polymeric additives in nucleic acid delivery formulations. Further studies are warranted to elucidate the underlying mechanisms of these effects and to optimize polymer inclusion for improved therapeutic outcomes.

### 3.7 LNP-mediated delivery of Luciferase mRNA guides the dose-dependent expression of Luciferase

**Fig. 5:**
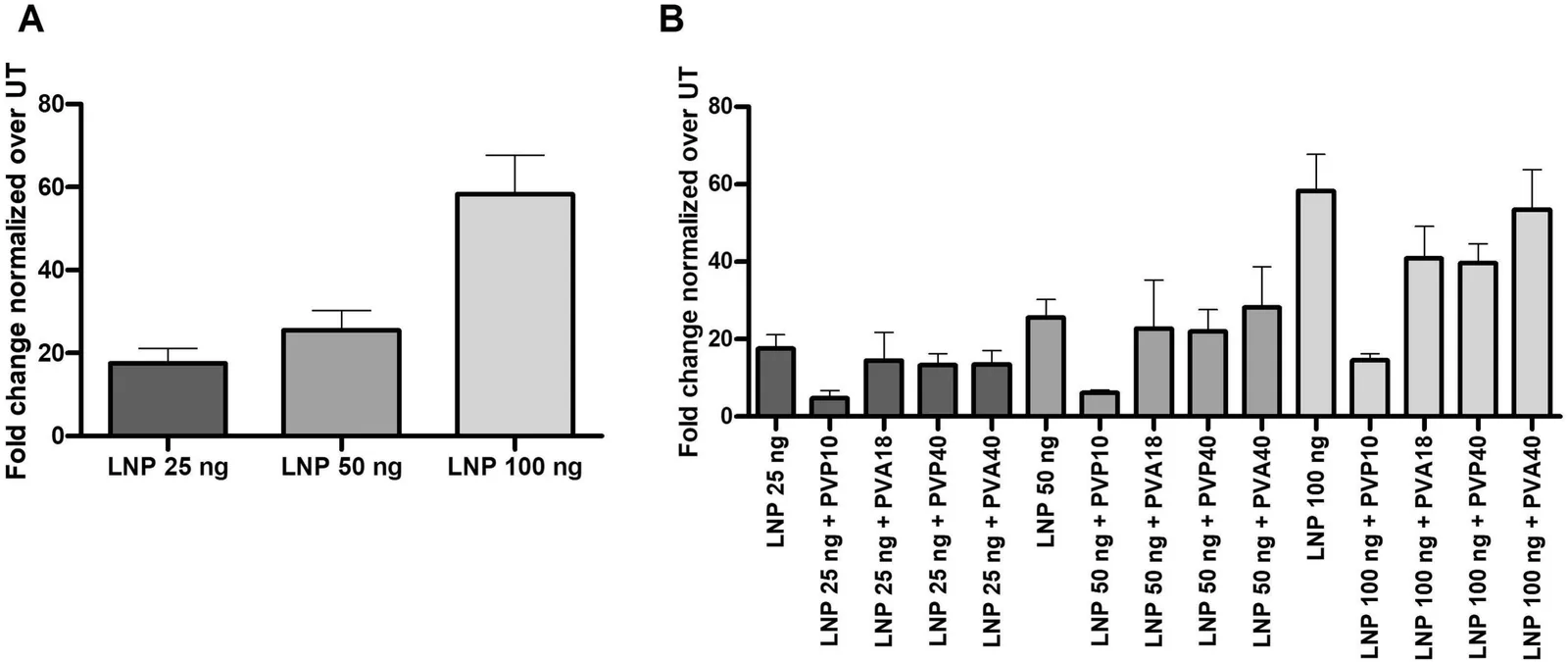
Luciferase Assay values after treatment with different doses of LNP containing Luciferase mRNA. Cells were treated with LNP formulations for 24 h. Thereafter, luciferase expression was measured by luminometric analysis. (A) LNPs were diluted in serum-free Opti-MEM media and then supplemented to the cells in three concentrations. (B) Diluted LNPs were supplemented with different polymers, and then these formulations were added to the cells in three concentrations to assess the effect on efficiency. Values indicate the mean of at least three independent experiments measured in triplicates (mean ± SEM, *N* ≥ 3). Statistical analyses (One-way ANOVA) were conducted with GraphPad Prism software, and using Bonferroni as a post-hoc test.

In the second phase of our study, we explored the application of LNPs for the efficient delivery of oligonucleotides, with a particular focus on mRNA. To evaluate this system, we transitioned from using the genetically modified HeLa 705 cell line to wild-type (WT) HeLa cells. The rationale behind this shift was to assess the efficacy of LNPs in delivering luciferase-encoding mRNA to unmodified cells, enabling a broader applicability of the results. To establish a dose-response relationship, we tested various concentrations of the mRNA by diluting the LNP stock in Opti-MEM. The luciferase signal, indicative of mRNA translation and protein activity, was found to be dose-dependent, with increasing signal intensities corresponding to higher mRNA concentrations (Fig. 5A). This result confirms the capacity of LNPs to facilitate efficient mRNA delivery in a concentration-dependent manner.

To examine whether polymer addition could further modulate LNP-mediated mRNA delivery, the same PVA and PVP excipients tested with PF14 complexes were added post-formulation to LNPs and evaluated across mRNA doses (Fig. 5B). High molecular weight polymers, particularly PVA40, enhanced luciferase expression compared to LNP alone, whereas low molecular weight PVP10 had no consistent enhancing effect. This pattern broadly mirrored the polymer dependence observed for PF14 complexes, suggesting that high-MW polymers may exert a generalizable stabilizing effect on nucleic acid delivery vehicles. As polymers were introduced after LNP formulation (Section 2.2), mRNA encapsulation was not affected by their addition.

Following the dose-response assessment, we focused on the condition corresponding to an mRNA concentration of 100 ng. This concentration was selected based on its ability to generate a robust luciferase signal while providing a practical framework for subsequent optimization and comparative studies. Future investigations will aim to refine LNP formulation parameters and explore their efficacy across diverse cell lines and experimental conditions, thereby contributing to the advancement of mRNA-based therapeutic delivery platforms.

### 3.8 Physical characterization confirms excipient-related changes in surface charges

**Fig. 6:**
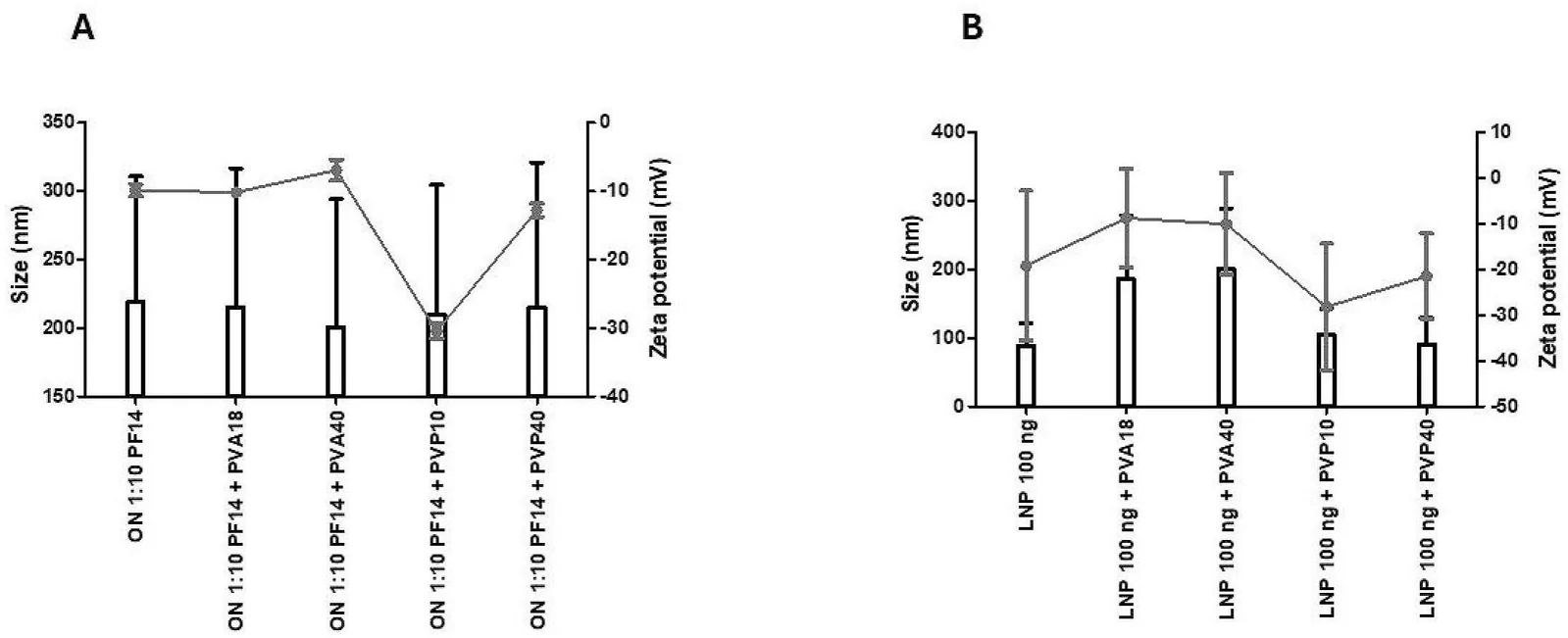
Size and zeta potential measurements of ON-705/PF14 and LNPs nanoparticles added with different polymers. Nanoparticles were analyzed using a ZetaView PMX system to assess particle size and zeta potential. ON-705/PF14 nanoparticles were formulated at a 1:10 MRs in serum-free Opti-MEM media (A), LNPs were diluted in serum-free Opti-MEM media (B), then both formulations were added with different polymers and diluted to perform the measurements. Values indicate the mean of at least three independent experiments measured in triplicates (mean ± SEM, *N* ≥ 3).

Following these findings, both the ON-705/PF14 and the LNPs formulations added with the different polymers, were measured using Zetaview, to determine changes in the size and electrical charge. For the ON-705-PF14 complexes (Fig. 6A), the addition of polymer excipients resulted in only minor changes in particle size, with values ranging from 200.8 ± 93.4 nm to 215.5 ± 101.1 nm for PVA- and PVP-containing formulations.

While particle size remained relatively stable, zeta potential varied depending on the excipient used. For example, the formulation containing PVP10 showed a more negative surface charge (−30.43 ± 1.07 mV) compared to the other polymer-containing formulations, which explains the lower efficacy of these complexes due to the high repulsion between the negative charges of the complexes and cellular membranes.

Regarding LNP formulations (Fig. 6B), they showed smaller particle sizes, with the control LNP displaying an average size of 88.2 ± 33.3 nm and a zeta potential of −19.17 ± 16.38 mV. The addition of excipients influenced particle size to different extents. Formulations containing PVA18 and PVA40 resulted in larger particles (∼186–200 nm), whereas PVP-containing formulations maintained smaller sizes (∼90–104 nm). Zeta potential values for the LNP formulations ranged from −8.76 ± 10.81 mV to −28.21 ± 13.83 mV, indicating variability in surface charge depending on the excipient used. We also noticed a correlation between the activity and lower negative charges, as in the case of PVA 18 and PVA 40 excipients.

Overall, the results demonstrate that the tested formulations produced nanoparticles within a size range suitable for nucleic acid delivery, while the polymer excipients primarily influenced surface charge rather than particle size.

### 3.9 Peptide/ON complexes and LNP-mediated delivery are inducing minimal toxicity

Two key requirements for an efficient delivery vector are high activity and low cytotoxicity. Because improved transfection performance can sometimes come at the expense of cell viability, we evaluated the toxicity of the different formulations in the HeLa Luc/705 reporter cell line under serum conditions.

**Fig. 7:**
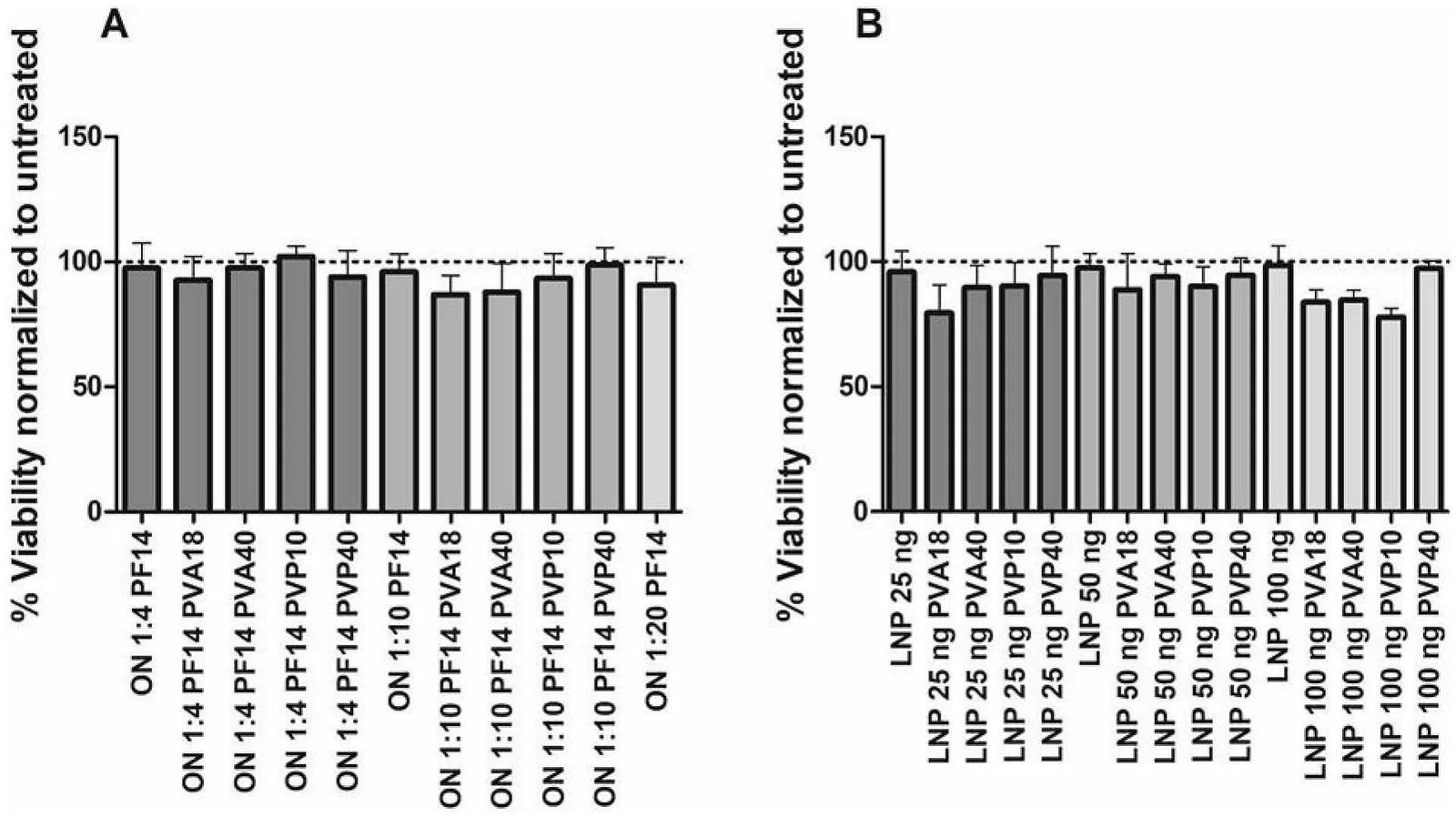
Viability measurements of different peptide/ON complexes and LNP treatments. A WST-1 assay was performed to assess cell viability after treatment. 24 h after treatment, the medium was replaced with fresh medium supplemented with WST-1 reagent and incubated at 37 °C for 2 h. Results are expressed as the percentage ratio of the absorbance at 450 nm of treated cells relative to untreated cells. (A) Viability after treatment with the ON-705/PF14 complexes. (B) Viability after treatment with the LNP formulations. Values indicate the mean of at least three independent experiments measured in triplicates (mean ± SEM, *N* ≥ 3). Statistical analyses (One-way ANOVA) were conducted with GraphPad Prism software, and using Bonferroni as a post-hoc test.

As shown in Fig. 7, the tested formulations are well tolerated and maintain cell viability at roughly 80–100% of untreated control levels, indicating that the complexes were generally well tolerated.

## 4 Discussion

This study systematically evaluated the key factors impacting the therapeutic efficacy of two promising nucleic acid delivery systems: PF14 complexes for SSOs and LNPs for mRNA delivery (Hou et al., 2021; Kowalski et al., 2019).

For PF14, increasing the peptide-to-oligonucleotide molar ratio enhanced cellular delivery and splice-switching activity up to a saturating ratio of 1:10. Fluorescence microscopy supported these findings by providing direct visual evidence of intracellular uptake of Alexa Fluor 568-labelled ON705. Uptake increased with increasing PF14:ON705 ratio and reached a plateau at 1:10, mirroring the trends observed in the luciferase assay. This enhancement likely reflects optimal nanoparticle formation, where sufficient peptide enables efficient cargo condensation, stability, and cellular uptake. The observed plateau suggests that further increases in peptide content do not overcome intrinsic limits in cellular uptake or intracellular processing. The progressive disappearance of free ON-705 with increasing PF14 ratio, observed by gel retardation, parallels the charge-neutralization trend seen by zeta potential, together providing physicochemical evidence that the plateau in luciferase signal at MR 1:10 corresponds to near-complete complexation of the oligonucleotide cargo.

Buffer composition significantly influenced complex stability and biological activity. Formulations in Opti-MEM consistently outperformed sugar-based buffers and conventional media. Physicochemical characterization (Fig. 3C) showed that complexes formed in Opti-MEM achieved less negative zeta potentials than those prepared in HBG, SH, RPMI, or DMEM, indicating more complete peptide–oligonucleotide charge neutralization. This likely supports more stable nanoparticle formation and improved cellular uptake under the tested conditions. Moreover, polymer effects were MR-dependent: PVA40 enhanced splice correction at MR 1:4, while PVA18 and PVP40 enhanced it at MR 1:10. Low molecular weight PVP consistently impaired efficacy across both ratios, likely reflecting its association with the more negative surface charge measured by zeta potential (Fig. 6A), which would increase electrostatic repulsion with the negatively charged cell membrane and thereby reduce cellular uptake. The mechanisms likely involve altered nanoparticle stability, reduced aggregation, or enhanced cellular interactions, warranting further biophysical characterization (Miao et al., 2021). In fact, the addition of PVA or PVP did not noticeably affect uptake of freshly prepared complexes, suggesting that the effects of these excipients on biological activity are more likely related to formulation stability than to changes in cellular internalization.

For LNP-based mRNA delivery, a clear dose-dependent luciferase expression was demonstrated, confirming the capacity of ionizable lipid nanoparticles for efficient mRNA transfection in mammalian cells. Addition of high molecular weight PVA and PVP polymers also augmented LNP-mediated mRNA expression, paralleling observations with PF14, suggesting that these polymers might generally stabilize delivery vehicles or promote better intracellular trafficking (Ezzat et al., 2010).

Collectively, these findings provide valuable insights for formulation strategies in nucleic acid therapeutics. Optimizing the peptide-to-oligonucleotide ratio, selecting suitable buffers, and incorporating stabilizing polymers are critical parameters for enhancing delivery efficiency and therapeutic outcomes. Further investigations including in vivo validations and mechanistic studies will be essential to translate these optimizations into clinical benefits.

One limitation we want to highlight is that although our cellular uptake and fluorescence microscopy data showed punctate cytoplasmic foci, which we initially interpreted as possible endosomal entrapment, this observation alone does not support firm conclusions regarding endosomal entrapment or increased internalization. We have therefore revised the claims to state that the images qualitatively suggest increased cell-associated fluorescence and complex uptake. Confirming endosomal entrapment would require confocal microscopy with nuclear and cytoplasmic counterstaining and an endosomal marker such as LysoTracker, as previously demonstrated for transportan 10-based delivery systems (de Mello et al., 2024). This was outside the scope of the present study, and further analysis is needed to clarify the mechanism of cellular entry and evaluate endosomal escape.

Another limitation of the present study is that ON-705 was employed primarily as a model splice-switching oligonucleotide to evaluate delivery performance. While this approach enabled systematic assessment of the delivery platform, it does not directly demonstrate therapeutic efficacy with clinically relevant nucleic acid cargos. Therefore, the translational potential of the system would be further strengthened by investigating the delivery of therapeutically relevant payloads, such as siRNA, mRNA, or genes with established anticancer activity. Future studies will focus on evaluating these cargos to validate the platform's applicability for cancer therapy and other nucleic acid-based therapeutic applications. Additionally, structural characterization of our nanoparticle formulations would provide valuable insight into their morphology and organization. Cryo-EM is particularly well-suited to soft-matter nanoparticles such as LNPs, and peptide complexes preserving their native hydrated structure without the artifacts introduced by conventional sample preparation (Biswas et al., 2025).

## 5 Conclusion

This study demonstrates that formulation parameters critically determine the efficiency of PF14-mediated SSO delivery and LNP-based mRNA delivery systems. We show that splice-switching activity is strongly dependent on peptide-to-oligonucleotide molar ratio and SSO dose, with a saturation point at a 1:10 ratio. Buffer composition and excipient selection further modulate delivery performance, with Opti-MEM providing optimal conditions and polymer molecular weight influencing efficacy in a formulation-dependent manner. In parallel, LNPs enabled robust, dose-dependent mRNA expression while maintaining low cytotoxicity. Physicochemical characterization revealed that variations in surface charge and particle size correlate with delivery efficiency. Together, these findings provide mechanistic insight and practical guidance for optimizing peptide- and lipid-based delivery systems, supporting the development of more effective nucleic acid therapeutics.

## Authors contribution

Experimental design and methodology: OS, ZMH, IES. Funding acquisition: OS, IES. Investigation: OS, DD, ZMH. Data analysis: OS, DD, IES. Supervision: OS. Writing original draft: OS, DD, IES. Manuscript review and editing: OS, DD, ZMH, IES.

## Authors declare that they have no competing financial interests in relation to the work described

The datasets generated during and/or analyzed during the current study are available from the corresponding author on reasonable request.

## CRediT authorship contribution statement

**Delia Daniele:** Writing – review & editing, Writing – original draft, Investigation, Data curation. **Zaw Myo Hein:** Writing – review & editing, Investigation, Conceptualization. **Osama Saher:** Writing – review & editing, Writing – original draft, Supervision, Investigation, Funding acquisition, Formal analysis, Conceptualization. **Ibrahim El-Serafi:** Writing – review & editing, Writing – original draft, Funding acquisition, Formal analysis, Conceptualization.

## Declaration of competing interest

Authors declare that they have no competing financial interests in relation to the work described.

## Data Availability

Data will be made available on request.
