# Supplementary material for: Optimizing nucleic acid delivery using PF14 peptide and lipid nanoparticle systems
Source: Int J Pharm X. 2026 Aug 21;12:100646. doi: 10.1016/j.ijpx.2026.100646 (PMC13524629; doi:10.1016/j.ijpx.2026.100646)
Supplement: Supplementary file 1 [file mmc1.docx]

**Optimizing Nucleic Acid Delivery Using PF14 Peptide and Lipid Nanoparticle Systems**

**Authors:** Delia Daniele^a,b,c^, Zaw Myo Hein^d^, Osama Saher^a,b,c,e*^, Ibrahim El-Serafi^d*^

^a^Department of Laboratory Medicine, Karolinska Institute, Stockholm, Sweden

^b^Department of Cellular Therapy and Allogeneic Stem Cell Transplantation (CAST), Karolinska University Hospital Huddinge and Karolinska Comprehensive Cancer Center, Stockholm, Sweden

^c^Karolinska ATMP Center, Karolinska Institutet, ANA Futura, Stockholm, Sweden

^d^Basic Medical Sciences Department, College of Medicine, Ajman University, Ajman, United Arab Emirates

^e^Department of Pharmaceutics and Industrial Pharmacy, Faculty of Pharmacy, Cairo University, Cairo, Egypt

^*^Authors contributed equally as last authors.

**Corresponding author:**

Dr. Osama Saher

Department of Laboratory Medicine, Karolinska Institute, Stockholm, Sweden

E-mail address: [osama.saher@ki.se](mailto:osama.saher@ki.se); ORCID: 0000-0003-3286-5706

Dr. Ibrahim El-Serafi

Basic Medical Sciences Department

College of Medicine, Ajman University, P.O. Box: 346, Ajman, UAE

Email: [i.elserafi@ajman.ac.ae](mailto:i.elserafi@ajman.ac.ae); ORCID: 0000-0002-0463-1518

Telephone: +971-6-705 5345, Mobile Phone: +971-50-1271447

**Supplementary Figure S1: The PF14:ON705 molar ratio governs cellular uptake and saturates at 1:10.**

Alexa Fluor 568-labelled ON705 was complexed with PepFect-14 (PF14) in Opti-MEM at molar ratios of from 1:1 to 1:20. Uptake was imaged after 4 h (upper row) and 24 h (lower row) on a Bio-Rad ZOE Fluorescent Cell Imager using the red channel (excitation 556/20 nm, emission 615/61 nm), with LED intensity, exposure, gain and contrast held constant across all conditions and timepoints. Scale bar, 100 µm.

**Supplementary Figure S2: Polymeric excipients do not alter uptake of PF14:ON705 complexes formed at a 1:10 molar ratio.**

Alexa Fluor 568-labelled ON705 was complexed with PF14 at a molar ratio of 1:10 with polyvinyl alcohol (PVA18, PVA40) or polyvinylpyrrolidone (PVP10, PVP40) excipients. Uptake was imaged after 4 h (upper row) and 24 h (lower row) on a Bio-Rad ZOE Fluorescent Cell Imager using the red channel (excitation 556/20 nm, emission 615/61 nm), with LED intensity, exposure, gain and contrast held constant across all conditions and timepoints. Scale bar, 100 µm.
